# Potentials of School Nursing for Strengthening the Health Literacy of Children, Parents and Teachers

**DOI:** 10.3390/ijerph17072577

**Published:** 2020-04-09

**Authors:** Elke de Buhr, Michael Ewers, Antje Tannen

**Affiliations:** 1 Berlin Institute of Health and Nursing Science, Charité-Universitätsmedizin Berlin, 13353 Berlin, Germany; michael.ewers@charite.de (M.E.); antje.tannen@charite.de (A.T.); 2 School of Public Health and Tropical Medicine, Tulane University of New Orleans, New Orleans, LA 70112, USA

**Keywords:** health literacy, school nursing, children, parents, teachers, school health

## Abstract

Health literacy (HL) plays a key role in explaining health disparities. School nurses (SN) provide health related expertise within the school setting. A positive effect on the HL of children but also their teachers and parents has been suggested by some research, but gaps persist in the available information. As a pilot project, SN, which are not common in German schools, were placed in 28 public elementary and secondary schools in two German states. Children (11+ years, n = 2773), parents (n = 3978) and teachers (n = 420) participated in a 2017 baseline (T0) survey. Data collection was repeated in 2018 (T1). HL was measured using the Health Literacy for School-Aged Children scale (HLSAC) (children) and the European Health Literacy Short Scale (HLS-EU-Q16) (adults). Descriptive and multivariate data analyses were carried out. The HL of all groups increased between T0 and T1. Low child HL decreased from 17.9% to 14.9%. Problematic and inadequate HL dropped from 43.8% to 38.8% among parents and from 49.9% to 45.8% among teachers. Improvements were significant for children and parents but not for the teachers. Despite the relatively short intervention period and a relatively non-specific spectrum of interventions, there is some evidence that SN may contribute to strengthening HL within the school setting. The longer-term effects of SN on health literacy and child health should be further examined. For this, a clearer conceptualization of the scope of work of the SN in Germany including their educational interventions is imperative.

## 1. Introduction

Health literacy, understood as the “people’s knowledge, motivation and competences to access, understand, appraise, and apply health information in order to make judgments and decisions in everyday life concerning healthcare, disease prevention and health promotion to maintain or improve quality of life during the life course” [1], plays a key role in explaining differences in health behaviors and outcomes across all population and age groups [2]. Health literacy is closely linked to health-related behavior, for instance, nutrition habits or physical activities [3,4,5]. Poor health literacy is correlated with more smoking, drinking and binge drinking, less physical activity, unhealthy nutritional habits, and higher risks in terms of sexual and injury behavior [3,4,5]. Good health literacy, on the other hand, is a major indicator of an individuals’ ability to make informed decisions about their lifestyle, diet, and health care and thus provides a good foundation for individual and population-related health promotion and disease prevention [6,7]

Efforts to improve health literacy are likely to have a greater impact if they start early, ideally while children and adolescents are developing their own health-related concepts and behaviors [8,9]. The ability to obtain, process, and understand health information will help them to seek health care, if needed, and to participate in health promotion and disease prevention measures. It is also known that poor health literacy of parents is associated with a reduced understanding of preventive care information, lower access to preventive care services and quite often with undesirable health exposures and outcomes for children including poor nutrition, obesity, poor oral health, exposure to second-hand tobacco smoke, higher rates of injury, mistakes in the administration of drugs, or unnecessary visits to emergency departments [10,11]. The health literacy of parents, families, and other people in the social context of children is critical for their health and wellbeing and needs to be taken into consideration.

National Action Plans in numerous countries recommend focusing on public schools as an ideal setting to enhance health literacy early in the lifespan [12,13,14], including in Germany [7,15]. However, public schools are often not well prepared to handle health related problems and promote health literacy or healthy behavior in the classroom or the broader school environment [16].

Sometimes teachers themselves show a limited level of health literacy and they may be uncertain how to support vulnerable children in their care such as chronically ill or disabled students [17,18]. These skills are becoming increasingly relevant since Germany started to accelerate the inclusion of chronically ill and disabled children into regular schools, in line with Article 24 of the UN Convention on the Rights of Persons with Disabilities (CRPD) [19]. Given this context, it is imperative to systematically strengthen the health literacy of students, parents, and teachers by building bridges between the educational and the health system through introducing health experts such as school nurses into the school setting. 

Internationally, school nurses work in public schools to provide health education, surveillance, and clinical services and to participate in setting-oriented public health and health promotion programs [20,21]. Specially trained nurses can conduct individualized and population-based health education, health promotion and health literacy enhancement for different target groups within the school setting [22]. They can also assess potential health risks of individuals and provide care to the acute or chronically ill and disabled, and thereby promote social inclusion of these groups into everyday school life [23,24,25]. 

Effective school nurses are highly trained professionals. They have a wide range of clinical, social, and educative competencies, combine nursing expertise, trustworthiness, presence and discretion, and can act as an advocate for children’s health concerns [18,21,26]. Compared with teachers, nurses do not assess learning achievements and therefore are more readily able to demonstrate “empathy, compassion, nurturance and uncritical acceptance” [27]. This may have a positive effect on the information seeking behavior of children and adolescents in their presence, and school nurses may serve as liaison between students, their families and teachers on the one hand and primary care and public health services on the other.

In their systematic review, Wainwright et al. concluded that the focus of research activities on school nurses tends to be either broad and role orientated or limited to specific aspects [27]. Studies indicate that school nurses who targeted specific populations have had some effects, e.g., on social inclusion [28], smoking cessation [29], TV viewing and physical activity [30] or management of selected chronic diseases, like asthma, and school absenteeism [31,32]. A positive impact of school nurses on the health literacy of children, parents and teachers is assumed in many policy papers and some authors also see positive influences of school nurses on the mental health literacy of students [33,34]. Still, the total number of studies on the effects of school nursing in the peer-reviewed literature remains small and school nursing activities have rarely been systematically evaluated [27,35].

In Germany, there are hardly any structures for school nursing and very few school nurses work in the country. The few that do are almost always employed by private (international) schools. To date, there is no standardized university-based training program for school or public health nurses in Germany. To potentially address this situation, a pilot project was carried out in the German federal states of Hessen (former West Germany) and Brandenburg (former East Germany). Adult and pediatric nurses, most of them with a traditional vocational training and some with years of clinical expertise, participated in an intensive three months education program followed by on-the job training while working as school nurses in 28 primary and secondary schools. During the intervention period, the school nurses provided health care for acute health problems and first aid, engaged in health promotion and prevention activities, supported students with chronic conditions and disabilities, and provided counseling and moderation [36]. While there were a number of defined tasks for the school nurses, including the expansion of health knowledge and health education, their terms of reference were not explicitly aimed at strengthening the health literacy in children or adults.

The pilot project was evaluated from 2016 to 2018 by the authors of this article using a comprehensive mixed-methods study design [18]. One of the main interests of the evaluation was the potential of school nurses to address health care needs within the school setting and to influence possible health related outcomes, such as the health behavior and health literacy primarily of students but also their parents and teachers. This article examines observed changes in the health literacy levels of children, parents and teachers at the selected schools and discusses and reflects on the role of school nurses in contributing to these changes.

## 2. Materials and Methods

Data were collected in the form of two cross-sectional surveys, a baseline survey (T0) and a follow-up survey (T1). The baseline survey was carried out within the first weeks after the school nurses started their work. A total of 28 elementary and secondary schools in Germany were included with some of the schools serving both elementary and secondary students (see Table 1).

Due to differences in the administrative processing, the intervention first started in Brandenburg, followed by Hessen. Each school nurse provided both primary care and health education at their assigned schools. They were not asked to follow a prescribed curriculum but prioritized their activities based on the school’s needs. Their time spent and the different activities they engaged in were documented. At the time of the follow-up survey the school nurses had been working approximately 9 (Hessen) to 14 (Brandenburg) months at the selected schools. Three distinct target populations were invited to participate in the surveys: (a) All children 11 years and older enrolled at the selected schools, (b) the parents of all children attending the selected schools, including children under 11 years, and (c) all teachers working at the selected schools, resulting in two independent cross-sectional samples (T0 in 2017 and T1 in 2018). Children under 11 years were not included in the survey since the self-administered questionnaire and health literacy assessment tool were not suitable for completion by young children. 

Child health literacy was measured using the Health Literacy for School-Aged Children (HLSAC) scale [37]. Grouped into questions assessing theoretical knowledge, practical knowledge, critical thinking, self-awareness, and citizenship, the HLSAC total score ranges from 10 (lowest) to 40 (highest) health literacy. The cross-national measurement invariance of the instrument was recently tested. In previous research, the HLSAC “exhibited high internal consistency (α = 0.85) and showed adequate fit with the data” and was found suitable for large-scale international health literacy studies on adolescents [38]. For our dataset, Cronbach´s Alpha coefficients ranged from 0.85 (T0) to 0.87 (T1). The questionnaire for children, adapting available tools from large epidemiological surveys [39,40,41], also covered socio-demographic information and health behaviors. Socioeconomic status (SES) was derived from a combination of parents’ educational level, vocational training, current position and income (“SES of household”). 

The health literacy of parents and teachers was assessed using the short version of the European Health Literacy Scale (HLS-EU-Q16), which measures the competencies of accessing, understanding, appraising and applying health-related information [1]. The reliability of the original scale’s 47 self-reported single items was found acceptable for the German version and reached Cronbach´s Alpha coefficients between 0.78 and 0.93 [42]. The HLS-EU-Q16 Short Scale, which was applied in this study, was derived using iterative item selection based on Rasch modeling as well as content and face validity criteria [43]. HLS-scores were measured on a 16-point scale with a minimum of 0 and a maximum of 16 points. The minimum of 0 points represents the least possible health literacy and the maximum of 16 points the highest possible score [39,44]. For our dataset, Cronbach´s Alpha coefficients ranged from 0.85 (T0) to 0.86 (T1).

The study received approval from the ethics committee of the German Society of Nursing Science on 19 July, 2017. Participation in the survey was voluntary and anonymous. The survey participants were provided with a written summary of the research objectives and methodology. Informed consent procedures were followed. All participating adults and any children 14 years and older provided written consent. Children below 14 years provided verbal assent. The voluntary nature and confidentiality of the assessment were explained to the younger age group, but they were not asked to sign a consent form. No child was included in the survey without the permission of their caregivers. All questionnaires were self-administered and a small pilot test was completed before the start of data collection, which was used to further refine the survey instruments. Teachers and parents were provided with a printed copy of their questionnaires, which were returned by a due date. The participating children completed their questionnaires in class during a period of time set aside for this task. They completed their questionnaires without assistance. The response rates were 36% in Brandenburg and 20% in Hessen for the child questionnaire, 35% in Brandenburg and 24% in Hessen for the parent questionnaire, and 46% in the Brandenburg and 26% in Hessen for the teacher questionnaire. The completed questionnaires were scanned and entered into an electronic database, followed by data cleaning. Health literacy scores were calculated for adults and children. Descriptive, bivariate, and multivariate analyses were carried out using SPSS 25 statistical software. Additional qualitative interviews were conducted with nurses and selected teachers, students, and parents. These interviews were analyzed using MAXQDA. 

## 3. Results

Among the children who completed the child questionnaire were 51% girls and 49% boys with an average age of 14 years. The sample of parents consisted primarily of mothers (>80%). The age of the parents ranged from 19 to 76 years with an average age of 38 years at T0 and 42 years at T1. Among the households that provided the required information, 1047 households (32.8%) had a high, 1358 households (42.6%) had a medium and 784 households (24.6%) had a low socio-economic status (T0). Both urban and rural schools were represented in the sample with 32.7% of the children attending school in cities above 100 thousand inhabitants, 37.3% attending school in cities between 20 and 100 thousand inhabitants, 21.7% attending school in towns between 10 and 20 thousand inhabitants, while fewer than 10% of the children attended schools in communities under 10 thousand inhabitants (T0). The sample of teachers also was mostly female (>75%) with an average age of 46.0 years at T0 and 44.8 years at T1. Most of the teachers were teaching at schools in Hessen (56.4%), while a smaller percentage (43.6%) taught at schools in Brandenburg (T0) (see Table 2 and Table 3). 

### 3.1. Health Literacy Matrix Single Items for Children

“Ability to give ideas on how to improve health in one’s immediate surroundings”, “ability to compare health-related information from different sources”, and “ability to decide if health-related information was right or wrong” were judged the most difficult of the HLSAC questionnaire items by the participating children at the time of T0 data collection with more than 30% indicating that for them this was “barely true” or “not at all true.” On the other hand, “ability to follow the instructions given by doctors and nurses”, “having good information regarding health”, and “ability to judge how one’s own behavior affects one’s health” were perceived as easier by the children with fewer than 20% indicating that they “barely” or “not at all” had this ability. The T1 results resemble the results from the earlier round of data collection but show an overall decrease in the percentage of children reporting difficulties (see Table 4).

### 3.2. Health Literacy Matrix Single Items for Parents

Finding “information on how to manage mental health problems like stress or depression” was judged to be “very difficult” by 6.4% of the parents at the time of T0 data collection and “fairly difficult” by an additional 34.8%. Judging “if the information on health risks in the media is reliable” was considered to be “very difficult” by 6.2% of the parents and “fairly difficult” by 44.0%. Also perceived as difficult was assessing “when you may need to get a second opinion from another doctor”, with 3.9% of respondents rating this task to be “very difficult” and 32.5% “fairly difficult.” In addition, the task of deciding “how you can protect yourself from illness based on information in the media” was also experienced as problematic by many parents, with 3.9% of the respondents reporting that this task was “very difficult” and 35.8% reporting it was “fairly difficult.” At the same time, following “instructions from your doctor or pharmacist”, understanding “your doctor’s or pharmacist’s instruction on how to take a prescribed medicine”, understanding “health warnings about behavior such as smoking, low physical activity and drinking too much”, and understanding “why you need health screenings” were considered to be much easier by the responding parents, with less than 10% considering these tasks to be “very difficult” or “fairly difficult.” Again, the T1 results show an overall decrease in the percentage of respondents reporting difficulties (see Table 5). 

### 3.3. Health Literacy Matrix Single Items for Teachers

Compared to the parents, the teachers expressed very similar challenges. Finding “information on how to manage mental health problems like stress or depression” was judged to be “very difficult” by 7.9% of the teachers at the time of T0 data collection and “fairly difficult” by an additional 40.9%. Judging “if the information on health risks in the media is reliable” was considered to be “very difficult” by 4.5% of the teachers and as “fairly difficult” by 47.1%. Also perceived as difficult was assessing “when you may need to get a second opinion from another doctor”, with 4.3% of respondents rating this task to be “very difficult” and 42.8% “fairly difficult.” In addition, the task of deciding “how you can protect yourself from illness based on information in the media” was also experienced as problematic by many teachers with 2.8% of the respondents reporting that this task was “very difficult” and 37.5% reporting it was “fairly difficult.” On the positive side, also mirroring the parents’ responses, a number of activities were perceived as comparatively easy, with under 10% of the teachers considering the following tasks to be very difficult” or “fairly difficult”: following “instructions from your doctor or pharmacist”, understanding “your doctor’s or pharmacist’s instruction on how to take a prescribed medicine”, understanding “health warnings about behavior such as smoking, low physical activity and drinking too much”, and understanding “why you need health screenings.” The T0 and T1 comparison of the single items for the teachers shows decreases in reported difficulty in some areas but increases in others (see Table 6). 

### 3.4. Level of Health Literacy and Proportion with Problematic or Inadequate Health Literacy

At the time of T0 data collection, 15.2% of the participating children showed a high level of health literacy (HLSAC scores 36–40, n = 347), 66.8% a medium level of health literacy (HLSAC scores 26–35, n = 1522), and 17.9% a low level of health literacy (HLSAC scores 10–25, n = 408). When the T1 data were collected, the percentage of children with high health literacy had increased to 19.2% while the percentage of children with medium and low health literacy had decreased. The difference between the T0 and the T1 measurement is significant with a chi square of 47.187 and *p* < 0.001 (see Table 7).

At T0 data collection, 56.2% of the responding parents showed sufficient health literacy (HLS-EU Q16 scores >12–16, n = 2098), 30.1% showed problematic health literacy (HLS-EU Q16 scores *>8–12*, n = 1122) and 13.7% showed inadequate health literacy (HLS-EU Q16 scores 0–8, n = 512). At T1 measurement, the percentage of parents with sufficient health literacy had increased to 61.2% while the percentage of parents with problematic and inadequate health literacy had decreased. Again, the difference between the T0 and the T1 measurement is significant with a chi square of 135.210 and *p* < 0.001 (see Table 8). 

The responding teachers also improved between the two times of data collection. While at the time of T0 data collection, 50.1% of the responding teachers showed sufficient health literacy (HLS-EU Q16 scores >12−16, n = 189), this percentage increased by 4.0% when the T1 data were collected. The percentage of teachers with problematic health literacy (HLS-EU Q16 scores >8−12, n = 148) decreased by 7% but those with inadequate health literacy (HLS-EU Q16 scores 0−8, n = 40) increased from 10.6% to 13.5%. Perhaps due to the small sample size, the difference between the T0 and the T1 measurement is not significant with a chi square of 7.017 and *p* > 0.5 (see Table 9).

## 4. Discussion

Promoting health literacy has become a “nursing imperative” [45] for all age groups but especially early in the lifespan and for students attending public schools. The activities of school nurses are not only aimed at improving school attendance by providing acute and clinical care but also at supporting the development of “knowledge, motivation and competences to access, understand, appraise, and apply health information in order to make judgments and take decisions in everyday life concerning healthcare, disease prevention and health promotion” [1,46]. 

Since the first school nurse projects have only recently been implemented in Germany, almost no data on possible outcomes for students, parents, and teachers were available at the start of the intervention [47]. Our research is the first study in Germany investigating the potential benefits of school nursing for the health literacy of students, parents, and teachers. The comparatively large sample of 28 primary and secondary schools including both urban and rural schools as well as schools in both East and West Germany approximate the diversity of the German educational landscape. Some limitations of the collected data include possible distortions due to the non-probability sampling of the selected schools, possible selection bias due to non-response, some differences in participation rates and sample composition at T0 and T1, possible recall and social desirability biases, and the lack of a comparison group. Furthermore, the school nursing interventions were not standardized, and some schools employed additional professionals such as psychologists or social workers. 

Despite these limitations, the study points to aspects of health literacy that are perceived as challenging by children, parents, and teachers. For the participating children the “ability to give ideas on how to improve health in one’s immediate surroundings” and “ability to compare health-related information from different sources” were the most difficult skills regarding health literacy. For the parents and teachers, finding information on mental health problems and judging health information in the media caused the main difficulties. These findings are in line with results from other European health literacy surveys [45]. The prevalence of problematic or inadequate health literacy among parents and teachers decreased from 43.8% to 38.8% (parents) and from 49.9% to 45.8% (teachers), with overall health literacy levels in these categories broadly comparable with previous estimates for adult populations in Germany and other European countries, which ranged from 29% to 62% [48].

For children, parents and teachers, the levels of health literacy improved between the T0 and T1 data collection. The observed changes could be driven by multiple factors. They could be directly impacted by social contacts between school nurses and students, parents and teachers in the school setting. It is also possible that the institutional change induced by the project led to more health awareness among all three populations. However, the relative contribution of the school nurses to changes in health literacy levels requires further investigation to rule out biases such as measurement effects caused by the repeated observations. Still, additional qualitative data from children, parents, and teachers collected as part of the intervention also point to benefits. These data indicate that the school nurses were accepted as experts in health questions at their schools and entrusted with tasks ranging from providing first aid and decision making after injuries to implementing diversified health promoting projects inside the classrooms. Findings from the qualitative interviews also show that the school nurses were regularly frequented as trusted representatives of the health sector to address individual health related questions and concerns [26], which may well have strengthened the ability of students and adults to seek, process, and understand basic health information resulting in more informed decisions and healthier behaviors. These findings are supported with the findings from other studies, which have also described students’ valuing the “empathy, compassion, nurturance and uncritical acceptance” of the nurses working at their schools [27]. 

Since Germany does not have an established school nursing tradition, very few schools have access to this resource. In the small number of schools that do have a school nurse, the emphasis is not always placed on educational interventions, counseling or other activities likely to directly contribute to strengthening the health literacy of children and adults due to numerous constraints including lack of time, inadequate training, and insufficient resources. The school nurses who participated in the pilot project were tasked with both the provision of primary care and involvement in health education and other health promotion activities. It is likely that the positive effects of school nurses on health literacy in the school setting could be further increased if the educational aspects of their work were expanded and their interventions more specific and standardized. Additional benefits could be expected if higher educated school nurses, ideally with a bachelor level education comparable to international standards, would be introduced. 

## 5. Conclusions

The concept of school nursing in public schools in Germany is evolving. After a late start, an increasing number of schools are benefiting from a school nurse. Moving forward, the professional profile of school nurses in Germany needs to be sharpened, strategies for creating a supportive and healthy environment need to be further developed, and more specific interventions for strengthening the health literacy of children, parents and teachers should be conceptualized, piloted and evaluated. The research findings provide first indications of possible benefits of school health nursing on the health literacy levels of primary and secondary school children, their parents and teachers. Future research should be directed at comparing intervention options and attributing change to specific activities further addressing concerns that “the evidence base relating to the impact of school nurses on the health of the school-age population is small and relatively weak” and “[m]odels for the assessment of the impact of school nursing on health outcomes and determinants of health require development” [35]. Based on the emerging evidence, the “new element” of school nurses could become a critical step towards creating health-literate organizations and healthy schools, in line with the whole school, whole community, whole child approach (WSCC) [46].

## Figures and Tables

**Table 1:** Type of schools included in sample.

Type of School	Brandenburg	Hessen
Elementary schools (primary schools)	12	
High schools (secondary schools)	5	8
Integrated schools (primary and secondary schools)	1	2
Total	18	10

**Table 2:** Sample characteristics (children, parents and teachers, T0 measurement, 2017).

Sample Characteristic	Children (11 Years and Older)	Parents	Teachers
Average age	14.2 years	38.1 years	46.0 years
Gender	Female: 51.7% Male: 48.3%	Mother: 81.9% Father: 17.0% Other: 1.1%	Female: 77.1% Male: 22.9%
N	2773	3978	420

**Table 3:** Sample characteristics (children, parents and teachers, T1 measurement, 2018).

Sample Characteristic	Children (11 Years and Older)	Parents	Teachers
Average age	14.1 years	42.0 years	44.8 years
Gender	Female: 51.0% Male: 49.0%	Mother: 83.2% Father: 16.1% Other: 0.7%	Female: 75.7% Male: 24.3%
N	2530	2503	477

**Table 4:** Health Literacy for School-Aged Children (HLSAC) Matrix Items (children 11 years and older, T0 and T1).

No.	Item	T0			T1		
		Not at All or Barely True ^1^	Absolutely or Somewhat True ^1^	N	Not at All or Barely True ^1^	Absolutely or Somewhat True ^1^	N
1	Having good information regarding health	16.5%	83.5%	2584	16.3%	83.7%	2560
2	Ability to give ideas on how to improve health in one’s immediate surroundings	33.9%	66.1%	2533	29.7%	70.3%	2525
3	Ability to compare health-related information from different sources	38.1%	61.9%	2596	32.6%	67.4%	2496
4	Ability to follow the instructions given by doctors and nurses	11.4%	88.6%	2543	11.8%	88.2%	2517
5	Ability to give examples of things that promote health	22.4%	77.6%	2527	19.7%	80.3%	2518
6	Ability to judge how one’s own actions affect the surrounding natural environment	22.5%	77.5%	2505	20.5%	79.5%	2493
7	Ability to find health-related information that is easy to understand	22.7%	77.3%	2496	20.1%	79.9%	2488
8	Ability to judge how one’s own behavior affects one’s health	19.3%	80.7%	2487	17.4%	82.6%	2491
9	Ability to decide if health-related information is right or wrong	31.1%	68.9%	2484	29.3%	70.7%	2493
10	Ability to justify one’s own choices regarding health	22.5%	77.5%	2482	19.9%	80.1%	2468

^1^ On a Scale from Absolutely True to Not at All True, Select the Most Accurate Response: ….

**Table 5:** European Health Literacy Short Scale (HLS-EU-Q16) Matrix Items (parents, T0 and T1).

No.	Item	T0			T1		
		Very or Fairly Difficult ^1^	Very or Fairly Easy ^1^	N	Very or Fairly Difficult ^1^	Very or Fairly Easy ^1^	N
1	Find information on treatments of illnesses that concern you?	24.0%	76.0%	3813	21.8%	78.2%	2425
2	Find out where to get professional help when you are ill?	20.5%	79.5%	3882	19.1%	80.9%	2475
3	Understand what your doctor says to you?	15.0%	85.0%	3920	14.1%	85.9%	2497
4	Understand your doctor’s or pharmacist’s instruction on how to take a prescribed medicine?	3.6%	96.4%	3943	4.0%	96.0%	2495
5	Judge when you may need to get a second opinion from another doctor?	36.4%	63.6%	3887	33.7%	66.3%	2467
6	Use information the doctor gives you to make decisions about your illness?	27.1%	72.9%	3853	24.3%	75.7%	2442
7	Follow instructions from your doctor or pharmacist?	4.8%	95.2%	3921	4.1%	95.9%	2513
8	Find information on how to manage mental health problems like stress or depression?	41.3%	58.7%	3731	40.1%	59.9%	2384
9	Understand health warnings about behavior such as smoking, low physical activity and drinking too much?	9.2%	90.8%	3849	7.8%	92.2%	2452
10	Understand why you need health screenings?	5.7%	94.3%	3895	4.5%	95.5%	2484
11	Judge if the information on health risks in the media is reliable?	50.2%	49.8%	3854	44.0%	56.0%	2453
12	Decide how you can protect yourself from illness based on information in the media?	39.7%	60.3%	3845	35.0%	65.0%	2457
13	Find out about activities that are good for your mental well-being?	31.5%	68.5%	3781	29.3%	70.7%	2410
14	Understand advice on health from family members or friends?	11.6%	88.4%	3849	11.0%	89.0%	2438
15	Understand information in the media on how to get healthier?	21.1%	78.9%	3827	18.9%	81.1%	2432
16	Judge which everyday behavior is related to your health?	17.3%	82.7%	3845	16.3%	83.7%	2429

^1^ On a Scale from Very Easy to Very Difficult, how Easy would you Say it is to: …

**Table 6:** HLS-EU-Q16 Health Literacy Matrix Items (teachers, T0 and T1).

No.	Item	T0			T1		
		Very or Fairly Difficult ^1^	Very or Fairly Easy ^1^	N	Very or Fairly Difficult ^1^	Very or Fairly Easy ^1^	N
1	Find information on treatments of illnesses that concern you?	23.8%	76.2%	387	26.7%	73.3%	442
2	Find out where to get professional help when you are ill?	26.3%	73.7%	396	23.8%	76.2%	445
3	Understand what your doctor says to you?	13.5%	86.5%	399	12.3%	87.7%	456
4	Understand your doctor’s or pharmacist’s instruction on how to take a prescribed medicine?	1.5%	98.5%	400	3.5%	96.5%	458
5	Judge when you may need to get a second opinion from another doctor?	47.1%	52.9%	395	45.3%	54.7%	455
6	Use information the doctor gives you to make decisions about your illness?	35.2%	64.8%	383	29.2%	70.8%	449
7	Follow instructions from your doctor or pharmacist?	5.3%	94.7%	399	4.8%	95.2%	456
8	Find information on how to manage mental health problems like stress or depression?	48.8%	51.2%	379	43.4%	56.6%	458
9	Understand health warnings about behavior such as smoking, low physical activity and drinking too much?	2.0%	98.0%	399	3.9%	96.1%	465
10	Understand why you need health screenings?	3.2%	96.8%	401	6.6%	93.4%	470
11	Judge if the information on health risks in the media is reliable?	51.6%	48.4%	399	57.9%	42.1%	461
12	Decide how you can protect yourself from illness based on information in the media?	40.3%	59.7%	395	42.0%	58.0%	455
13	Find out about activities that are good for your mental well-being?	23.2%	76.8%	393	28.9%	71.1%	443
14	Understand advice on health from family members or friends?	9.8%	90.2%	396	10.7%	89.3%	467
15	Understand information in the media on how to get healthier?	18.7%	81.3%	390	16.5%	83.5%	467
16	Judge which everyday behavior is related to your health?	15.1%	84.9%	398	14.3%	85.7%	454

^1^ On a Scale from Very Easy to Very Difficult, how Easy would you Say it is to: ….

**Table 7:** Proportion of children (11 years and older) with different levels health literacy.

Health Literacy Level	Percentage (T0)	Percentage (T1)	Difference	Chi Square N = 316	Cramer’s V N = 316
High	15.2	19.2	+4.0		
Medium	66.8	65.9	−0.9	47.187 ***	0.273 ***
Low	17.9	14.9	−3.0		

Low health literacy ranging from 10–25, medium health literacy ranging from 26–35, and high health literacy ranging from 36-40 based on HLSAC. *** Results significant at the 0.001 level.

**Table 8:** Proportion of parents with different levels of health literacy.

Health Literacy Level	Percentage (T0)	Percentage (T1)	Difference	Chi Square N = 588	Cramer’s V N = 588
Sufficient	56.2	61.2	+5.0		
Problematic Inadequate	30.1 13.7	25.9 12.9	−4.2 −0.8	135.210 ***	0.339 ***

Inadequate health literacy ranging from 0−8, problematic health literacy ranging from >8−12, sufficient health literacy ranging from >12−16 based on HLS-EU Q16. *** Results significant at the 0.001 level.

**Table 9:** Proportion of teachers with different levels of health literacy.

Health Literacy Level	Percentage (T0)	Percentage (T1)	Difference	Chi Square N = 44	Cramer’s V N = 44
Sufficient	50.1	54.1	+4.0		
Problematic Inadequate	39.3 10.6	32.3 13.5	−7.0 +2.9	7.017	0.282

Inadequate health literacy ranging from 0–8, problematic health literacy ranging from >8–12, sufficient health literacy ranging from >12–16 based on HLS-EU Q16.
